# Hints on the Morality of Medicine

**Published:** 1831

**Authors:** Thomas D. Mitchell

**Affiliations:** Professor of Chemistry and Pharmacy, in Miami University


Art. II. *Hints on the Morality of Medicine.*—By Thomas D. Mitchell, M. D., *Professor of Chemistry and Pharmacy, in Miami University.*

Of all the arts and professions in society, we are aware of none, in which improvement has been so great and unceasing, as in the profession of medicine. In all the departments, the hand of discovery has been constantly at work; and in modern times, the most laborious investigations and the most untiring researches have been pursued, in order, if possible, to elevate the science, as a whole, to the highest order of perfection. Let us glance for a moment, by way of illustration, at the almost magical operations of chemical analysis. We there behold the finest specimens of accuracy and scrutinizing inquiry, that are to be seen in any of the departments of human science. Results have been obtained, at which no one, a century ago, had ventured to direct even his thoughts, and although among the reputed discoveries of an eminent modern philosopher, there are some to which he has only a questionable claim, yet enough is to be seen, resting on the sure and unmovable basis of fact, to justify our assertion in respect of the steady, onward march of medical science. For although the chemical researches of recent date, may be fitly ranked among the trophies of the science of chemistry, the ultimate tendency of all them is to give perfection to the art of healing. As evidence to this point, I have only to refer the reader to the labors of Sertuerner, Robiquet, and others in France, and Carpenter, Staples, &c., in the United States of America. These gentlemen have furnished some very powerful auxiliaries in the remedial department of our science, as, for instance, cornine, salicine, morphia, emetine, and others, which I need not here enumerate.

Similar remarks might be made in relation to botanical improvements, and to the advances of every variety of medical investigation; and the conclusion from the whole would be, that the legitimate end of all this diversified research, is the perfection of the medical profession, as a unit.

But, however diversified, incessant, and laborious the exertions of scientific men may be, for the attainment of the great object in view, and notwithstanding the apparent elevation which our profession acquires from these sources, something additional is requisite. The edifice may be grand; its walls may be firm as adamant, and its foundation unyielding as the rock; but it is unfinished still. There is a deficiency within and without, which neither chemistry, nor botany, nor anatomy, nor any branch of science, ordinarily taught in medical schools, has the power to supply. And it is the design of this paper, in a very brief manner, to set forth the proper remedy for this defect.

By the morality of medicine, we simply mean, the exemplification of all the essential principles of good morals and virtuous conduct in the practice in the art of healing. We intend to say, and wish so to be understood, that a vicious and immoral man, cannot be a truly good physician, even though his attainments exceeded those of Boerhaave, and his fame outstripped the reputation of Hippocrates. We are aware, that such an assertion comes in direct collission with the common parlance of society, and is at variance with the prevalent notions of every community. But were these objections of any value. they might be brought to bear with mighty force, against all the finer feelings of the human breast, and their ultimate tendency would be, to turn back the tide of civilization, and to restore the crown to savage life.

Our intention, on the present occasion, is to consider but three of the features of the morality of medicine, and these will be found to include, essentially, all that is important in the subject; they are *veracity*, *temperance*, and a *respect for divine institutions*.

First, of *veracity*. And here I may premise, that the physician, whose conduct is evidential of a strict regard for veracity, as based on the solid foundation of true virtue, cannot fail to exhibit in his deportment, the other items of professional morality. But, there is among medical men, so lamentable a destitution of the points of character already cited, that it is needful, to be somewhat specific in the examination of these topics. Nor do I dream of doing a service, in this feeble effort, to any who have grown grey in the practice of bad habits; they will not condescend to read any remarks, or if they do, will pronounce them foolish and weak. I am not, however, in despair respecting the juniors of our profession, and of those who are about to enter upon its important and arduous duties; to them, in an especial manner, are these hints directed.

The advantages of veracity may be discovered from negative as well as from positive sources. And we can, in no way, so well appreciate its importance, as by taking a survey of the evils resulting from a neglect or destitution of this moral quality. The physician, who cares not for his word, whether he be found in the private walks of practice, or in the public arena, as a teacher, very soon finds his name associated with epithets of disrespect, and his integrity becomes so intangible, that it is difficult to determine whether it has any real existence or not. We sometimes witness a disregard for veracity, in statements of cases of disease, which never had any other than an imaginary form. Various motives often prompt medical men to talk and write about strange and inexplicable morbid phenomena, the creatures of their fancy, which, were they permitted to occupy their legitimate place, might prove very harmless tenants; but the inventors, desirous of reaping a little fame, at all risks, must forsooth tell out their story, and that is not the worst feature of the case; there are some persons sufficiently credulous to believe the well dressed fabrication. I know more than one individual, who, if fame tell the truth, always has a case at hand, to justify his mode of practice, and if one will not suffice, his inventive mind readily furnishes a second and a third, and as many more as the circumstances may seem to require. The result of this foolish and utterly ridiculous practice, is not dissimilar from the fabled fate of the boy and the wolf. Ultimately, all confidence is lost, and that which, in other hands, might occasionally produce much effect, is here a matter of entire waste.

The habit of falsifying has tended more than any other cause, to produce strife and bad feeling in the medical brotherhood. I do not believe that any serious difficulty ever existed among professional men, in any village or city, that had not for its principal source, a disregard for veracity. This has been seen in the frequent exaggerations of stories, and the cameleon-like gloss which has been put upon them, which in their simple and unvarnished state, were altogether harmless. The professed and disciplined tale-bearer cannot long possess the *esprit du corps*, without a departure from the law of veracity; and hence we find universally associated, the love of tattle and the lack of this fundamental principle in good morals.

The want of veracity often lays the foundation for that neglect of punctuality in consultations, which has extorted from some of the veterans in our profession, such loud and repeated complaints. "Every hour of my time," said an eminent professor in Pennsylvania, "is worth a guinea, and I cannot agree to consult with men who value time at so cheap a rate, as to oblige me to wait their convenience a full half hour beyond the specified period. Such neglect is not simply a positive loss to me, but it is an evidence of the want of interest in the particular case." Another professor, in one of his valedictory addresses, cited the case of an English nobleman, to enforce the duty of punctuality. The nobleman remarked to a friend, somewhat in the following strain; "so utterly do I abhor and detest the least semblance of falsifying, that if I had made an engagement with a peasant to play a game at push-pin, in the remotest corner of my forest, at a specified moment, no earthly consideration should induce me to fail in the strict fulfilment of the contract." And, if in a matter of so little moment, a nobleman could thus feel and act, in what regard should a physician hold his solemn promise to meet a professional brother at a stipulated point of time, to consult on matters involving all that is dear in life, or terrible in death? I know that circumstances may occur, to prevent the punctual observance of the consultation hour, and I most cheerfully make allowances for them. But when a reckless negligence of all the rules of propriety in these matters is apparent, when the evil complained of is habitual, the plea of circumstances is out of the question and goes for nothing; and I fear it will be found to be a truth, with very few exceptions, that the man who makes shipwreck of his veracity in the important business of consultation, has made a voluntary abandonment of this finest feature in medical ethics.

Appalling evils have found entrance into *medical schools*, by reason of departures from the sacred obligations of truth, and some of these institutions, whose dawning day was bright with hope and indicative of ultimate success, have sunk beneath a cloud, whence the thunder and lightning of violated faith have issued, to do the work of ruin and desolation. A single individual in a medical school, who is regardless of the high and solemn sanctions of truth, may and will, if providence interfere not, bring anarchy and overthrow upon the most prosperous establishment. Who then can remain unconcerned in a matter of such momentous consequence? If the want of veracity be, in reality, so prolific of evils to individual physicians, and to associates of Medical men, is it not of the last importance, to enforce, by every suitable argument, the indispensable necessity of this virtue, upon those who are preparing to enter the temple of Esculapius, but who have not yet passed its threshold? I call upon those who occupy the responsible station of preceptors, whether private or public, to consider well the nature of their duty. On them, to a great extent, rests the formation of Medical characters, and they must answer at the bar of public opinion, if no where else, for the faithful, upright discharge of the trust committed to their hands.

It were easy to enlarge on this highly interesting subject, and to show the importance of veracity, in its *positive* operations; but as I am desirous to edify the reader, rather than to weary his patience, I shall pass to the second topic named in the commencement of this paper.

*Of Temperance.* This term, in its general acceptation, has reference to the subjection of every inordinate desire, or propensity to which human nature is prone. Every practice or habit, the effect of which is to derange the physical or mental operations, and so unfit the man for the ordinary business of life, and especially for professional duty, is at war with the fundamental principles of temperance. Hence not only is excess in eating or drinking, a violation of the law of temperance, but the indulgence of malignant passions, and the withholding from nature her just claims of proper hours for repose, by intense study, are alike transgressions of this moral statute. But my design, at present, is to expose the odious vice of intemperance in the use of intoxicating liquors; because, more than any other or than all other departures from the principles of temperance, this vice degrades and brutalizes the physician, who sinks beneath its fearful influence.

It is matter of congratulation, that so many worthy members of our profession, have taken a decided stand in favor of the most rigid temperance. All that is requisite in order to complete success, is to subvert custom. Let physicians of high standing and exalted rank establish the fashion in this thing, and the work of reformation will be accomplished with facility. the combined influence of one hundred leading men in the profession of medicine, would suffice to cover, with enduring infamy, the worse than savage crime of drunkenness.

To effect a reformation in this matter, more is requisite than the simple writing or delivery of addresses. The force of example is no where so conspicuous as here, and the physician who would acquire a reputation for consistency in the matter of temperance, will not only abstain from the personal use of intoxicating liquors, but will make every laudable effort to expunge them from the Materia Medica. Well do I remember the emphatic manner in which a public medical teacher charged upon our profession the crime of making drunkards by wholesale, as the result of alcoholic potations prescribed for the cure of intermittents, who himself fell a martyr to intemperance. Like too many in the present day, his precept and example were the antipodes of each other.

But the work of reformation, as respects the medical profession, must have its beginning in early life. The student must be impressed with the importance of temperance, at the commencement of his enterprise. I would not, for all the wealth of these United States, be accessary to the introduction of a young man within the pale of our profession, who had imbibed a love for intoxicating liquors. Were his talents and acquirements of the highest order, his deportment, in general, amiable, and his connexions in life of the most respectable sort, this solitary blemish would mar the whole, in my estimation. To all young men, who have the profession of medicine in view, I would address the warning voice and friendly counsel, in this critical matter. Beware of intoxicating liquors, in whatever shape they meet your eye. They have within them the elements of destruction, infamy, and death. As you would escape the frightful storm that gathers in the distant sky, and which shall presently beat with relentless fury on the unsheltered traveller; so I beseech you, shun the intoxicating cup, whose poison is daily and hourly prostrating in the grave, the finest talents and the fairest prospects.

It may not be unavailing to reiterate a remark already made in reference to the expurgation of the Materia Medica of spirituous formulæ. Professor Sewall, of the District of Columbia, has taken an honorable stand in this aspect of the temperance reformation; and the writer of this paper has made a humble contribution in the same cause, as may be seen by reference to the Journal of Humanity, for the year 1830. Sufficient evidence has thus been accumulated to show, that if alcoholic medicines cannot be entirely excluded, at least, a very large portion may be dispensed with, in the treatment of disease. I firmly believe, that a combination of medical men, assisted by a suitable apothecary, could effect such a revolution in the Materia Medica, as would ultimately redeem our profession from the odium that has so long and so justly rested upon it. I have made the experiment, single handed, on a small scale, and the result has been, that not one spirituous article now in use as an internal remedy, is indispensable in the successful practice of medicine. And if this persuasion could be made general, it would tend powerfully to the promotion of temperance among medical men. For the constant handling of tinctures, a circumstance peculiar to country practitioners, leads to the occasional and frequent use of such of them, as are not absolutely unpleasant or disgusting to the taste; and hence it is, that intemperance is more frequent among physicians in country situations, than among city practitoners, who are not compelled to compound their own prescriptions.

I am aware of the errors in public judgment, respecting intemperate physicians, and have seen, with regret and astonishment, some well-informed and influential men, bestow their patronage on practitioners, who had become habitual drunkards. And I know, too, that intemperate physicians almost always leave behind them, at least, one legacy for the benefit of society, and that is, a character for pre-eminent skill. Who ever heard a tea-table history of such a physician, that did not embrace a high-toned commendation? "Poor fellow, he had but one fault, but yet he was the greatest physician, the most skilful doctor that ever practised in our village." But the feebleness of human discernment in these matters, offers no solid argument against the position we are defending; and should rather operate as an incentive, to retrieve the profession from the disgrace that intemperance has brought upon it.

In a discourse on the medical character, published in the New-York Medical Repository, about fourteen years ago, I proposed that all Universities and Colleges, conferring the degree of Doctor of Medicine, should be invested with retrospective powers; that in addition to their right of judging of the qualifications of candidates for degress, they should also exercise a supervision of all, who, from time to time, had the honors of the school conferred upon them. In the discharge of this duty, it was suggested, as highly proper, that the authority to practice medicine, which had been given in consequence of the exhibition of suitable talents and acquirements, should be taken away entirely, or suspended for a time, when palpable evidence manifested a disqualification for professional services, arising out of criminal conduct or vicious deportment, of any sort whatever. My opinion in this matter is not changed, but confirmed; and I humbly submit to the consideration of my brethren, whether such a provision would not be better calculated to expel intemperance from our ranks, than any other that has been proposed. It might fail, I grant, to cure the individual of his moral malady, but it would, most certainly, rid the fraternity of all the habitual intemperance that now fastens on it, and, by the public examples thus made, it might prove a salutary warning to the juniors of the profession.

The last item to be noticed in the consideration of the *Morality of Medicine*, is *respect for divine institutions*. And in briefly examining this important subject, I shall restrict myself to two vices, only, both of which are prevalent, to a considerable extent, among the members of our profession; they are, *profanity*, and *disregard for the Christian Sabbath.*

In relation to the charge of profanity, so often preferred against medical men, we may be permitted to say, that fashion, or perhaps a better motive, has operated to make the crime much more odious in professional estimation, than it was formerly considered. But there is still much ground to justify the allegations so frequently repeated, and it becomes every lover of sound ethics, to act vigorously in order to extirpate every root of so noxious and unsightly a weed.

There was a time in this country, when moral, or rather immoral monstrosities, were admired, and when hard swearing and profane jest, seemed to serve the purpose of unfailing commendation. A physician in an eastern city, gained an immense practice and an irresistible influence, chiefly from this source. He flourished, it is true, nearly eighty years ago, when refined manners were not so fashionable as in the present day. His was not the occasional or accidental oath, but the continued blast of vulgar profanity, which, while it set all human courtesy at defiance, poured contempt and malignity on the divine character. Nor did his popularity rest merely on the patronage of men like himself, but some who held his immoralities in utter disgust, were weak enough to seek his aid in the hour of sickness. I have heard this individual's name quoted, as proof, that it was of no consequence, what a man's moral character was, provided he was master of his profession; and this is the sentiment of many persons in the present day. It is presumable, however, that such conduct can receive the approbation of no one, whose moral sensibilities are not blunted, and whose taste is not morbid in the extreme. Indeed, posterity has judged rightly of the individual just referred to, and made some amends for the foolish and perverted judgment of a misguided ancestry. His name is seldom mentioned, without a simultaneous association with all that was depraved and savage in his character. Let those who would justify a similar deportment, by reference to so odious a precedent, give to the entire history its legitimate application, and we believe, that, instead of pursuing the course of profanity, in the expectation of reaping the same professional success which marked the career of their exemplar, they would shun his beaten path, fearful of the honest verdict of after times, which, on all their ephemeral glory, would write infamy and disgrace.

I know that a good deal of moral courage is required, to enable a young student of medicine to resist the manly example, as some call it, of his fellows; and hence it is, that the vice of swearing is so common in medical classes. Some there are, who call themselves men of honor, and who would not refuse a challenge to fight a duel, who are too dastardly to look puerile impudence and arrogance in the face, and to frown from their presence, the foul and ungentlemanly oath. The young man who furnishes evidence of bravery, sufficient to meet such an emergency, by a deportment which virtue only can inspire and maintain on a solid basis, has infinitely more, and better fortitude, than the profane and profligate duellist, who has killed his man, once and again. It cannot be supposed, that any man becomes a swearer from the mere love of profanity, or that, in his calm and reflecting hours, he can obtain the acquiescence of his judgment, in respect of such gross derilections from the path of rectitude. We envy not the moral darkness and the mental torpor of that profane individual, whose solitude is not occasionally disturbed with harassing thoughts and appalling spectres. And yet, it is conceivable, that a man may so fasten the vulgar habit of swearing upon himself, as to be almost insensible of the practice, until the custom shall seem to constitute a law of his very nature.

There is no class in society, excepting the ministers of the gospel,* on whom habits of profanity sit with so ill grace, as on physicians. Next to the clergy, their example and influence is regarded, in all that is dear in social and domestic life. They operate in their respective spheres of duty, on families and individuals, with an irresistible power, and many characters are predicated of their example. Surely, then, it is matter of deep importance, not only as respects the dignity of our profession, but in regard to probable influences on the moral aspect of society, that medical men should, by precept and example, evince their utter abhorrence of so debasing a vice.

In very near relation to the crime of profanity, stands the allegation of neglect and contempt of the Christian Sabbath, and of all religious exercises connected therewith. I am conscious, that in noticing this moral defect, I do not proceed on ex parte testimony; for my own early history, like that of almost all students of medicine, furnishes the total amount of evidence that can be offered, in defence or justification. Engaged in the

* We intend no reflection on the clergy, although it has been our misfortune to know more than one, who was *called* a minister, who was profane.

prosecution of studies, whose legitimate tendency is to inspire the mind with exalted views of Deity, and which proclaim, with mighty force, the doctrine of an intelligent, superintending Providence, the vanity of the merest stripling leads him to the idiotism of atheistic folly; and, bloated with a degree of self-importance, that evinces the actual imbecillity of the man, he repudiates the authority of Heaven, and sneers at all the institutions of the Christian religion. Nor does he regard, as of any value, the testimony of those who once thought as he does, but who have been induced to change their views, and to reform their practice. They, too, in his estimation, are weak-minded, fanatical, and the easy dupes of superstition and priest-craft, while he, forsooth, is wiser than Solomon, and carries the key which opens the temple of knowledge, in his right hand. I know not whether to pity or despise the frothy ebullitions of a half educated adventurer in medicine, who, puffed out with self-esteem, almost to the point of bursting, would force the ignorant to believe, that it were an easy thing to make a world; and that to regulate the operations of Nature, neither law nor intelligence were necessary. Yet such philosophers there are; and in their wisdom, they cast away the restraints which religion imposes, and choose to be governed by no authority, unless it be *chance.*

But it is matter of rejoicing, that our profession boasts of other philosophers than these; of men whose names and virtues shall flourish in the bloom of youth and beauty, while the memory of the atheist shall be blotted out, or engraved on the records of infamy. Yes, we do rejoice in the truth, that Sydenham, and Boerhaave, and Fothergill, and Locke, and Rush, and Good, were advocates of the Christian religion; and, standing behind the bulwark which their names have assisted in forming, the onsets of the enemies of good order, can do no harm, and should, therefore, excite no apprehensions.

The importance of the Christian Sabbath, not less as a civil than a moral institution, is so generally admitted in society, that it were a sort of moral treason, openly to reject it; and the code of laws which fashion has formed, is so modified, as to give, at least, a silent consent to its authority. And, let me ask, what can a physician lose, by a strict external regard to this institution; and on the other hand, may he not be a gainer, by a respectful observance of the duties connected with it? The testimony of one of the most learned men in another profession, has been often adduced, in the consideration of the matter before us, and must ever be in point. That ornament of the law, Sir Matthew Hale, long ago declared, that just in proportion to his strict observance of the Christian Sabbath, was his success in all the business of life; and men of the highest professional eminence in our own country, have borne repeated testimony to the same cardinal truth. So deeply am I impressed with the importance of this subject, that I do not hesitate to affirm, that the physician who neglects or contemns the institutions of religion, has no right to calculate on professional success, however great his attainments, or vigorous his powers may be; although I will not say, that some grossly immoral practitioners have not grown wealthy. But let it be recollected, that to amass wealth by practice, is a different thing from the acquisition of professional eminence and success. He whose talents and virtues have elevated him to an exalted station in the view of his brethren and of society, although he die a bankrupt, leaves behind him a more valuable treasure, than the avaricious and penurious practitioner can possibly bequeath, who cares not for laws, human or divine, but who glories in his stocks, and houses, and lands, and in them only.

Let the student of medicine seriously consider this subject. His future prospects, his character in society, will correspond, in all probability, with the moral sense which he is now acquiring. This is the crisis; the turning point in his history; his destiny hangs upon it. And when men in high places, set before him the pattern of immorality and infidelity, let him not forget the names of the *greatest among the great*, who, while they shine in the firmament of science, have left behind them an imperishable testimony in favor of virtue and good order.

